# First Comprehensive Proteome Analyses of Lysine Acetylation and Succinylation in Seedling Leaves of *Brachypodium distachyon* L.

**DOI:** 10.1038/srep31576

**Published:** 2016-08-12

**Authors:** Shoumin Zhen, Xiong Deng, Jian Wang, Gengrui Zhu, Hui Cao, Linlin Yuan, Yueming Yan

**Affiliations:** 1 College of Life Science, Capital Normal University, Beijing, 100048, China

## Abstract

Protein acetylation and succinylation are the most crucial protein post-translational modifications (PTMs) involved in the regulation of plant growth and development. In this study, we present the first lysine-acetylation and lysine-succinylation proteome analysis of seedling leaves in *Brachypodium distachyon* L (Bd). Using high accuracy nano LC-MS/MS combined with affinity purification, we identified a total of 636 lysine-acetylated sites in 353 proteins and 605 lysine-succinylated sites in 262 proteins. These proteins participated in many biology processes, with various molecular functions. In particular, 119 proteins and 115 sites were found to be both acetylated and succinylated, simultaneously. Among the 353 acetylated proteins, 148 had acetylation orthologs in *Oryza sativa* L., *Arabidopsis thaliana*, *Synechocystis* sp. PCC 6803, and *Glycine max* L. Among the 262 succinylated proteins, 170 of them were found to have homologous proteins in *Oryza sativa* L., *Escherichia coli*, *Sacchayromyces cerevisiae*, or *Homo sapiens*. Motif-X analysis of the acetylated and succinylated sites identified two new acetylated motifs (K---K and K-I-K) and twelve significantly enriched succinylated motifs for the first time, which could serve as possible binding loci for future studies in plants. Our comprehensive dataset provides a promising starting point for further functional analysis of acetylation and succinylation in Bd and other plant species.

*Brachypodium distachyon* L., endemic to the Mediterranean and Middle East, is a member of the Pooideae subfamily and a temperate wild annual grass^1^. It has rapidly become a model plant, especially for economically important crops with large genomes, such as wheat (*Triticum aestivum* L.), and several potential biofuel grasses such as switchgrass. *Brachypodium distachyon* accession 21 (Bd21) offers many advantages, such as a small diploid genome (272 Mbp), self-fertility, simple nutrient requirements for research^2^, and competence to be transformed efficiently^3^. Sequencing and annotation of the Bd21 genome were recently completed^1^, making further functional proteomic studies feasible.

Protein regulation encompasses multilayered and interconnected transcriptional and translational processes^4^. Subsequent protein PTMs allow for regulating protein function. Many PTMs are reversible and modulate the localization, activity, turnover, and interactions of proteins^5^. To date, more than 461 distinct PTMs have been described^6^ and it is increasingly clear that many, perhaps most, proteins have multiple PTMs^7^ created by processes such as ubiquitination^8^, SUMOylation^9^, acetylation^10^, and succinylation^11^. Among these PTMs, Lys-N^ε^-acetylation (PKA) has recently been shown to be nearly as abundant as O-phosphorylation^12^, and a prevalent modification in enzymes that catalyze intermediate metabolism.

Virtually all enzymes in glycolysis, gluconeogenesis, the tricarboxylic acid (TCA) cycle, and glycogen metabolism were found to be acetylated^13^, demonstrating the important roles of lysine acetylation (Kac) in various metabolic pathways. In addition, accumulating evidence suggests that lysine succinylation (Ksuc) is a widespread and important PTM in both eukaryotes and prokaryotes, and regulates diverse cellular processes^14^. Thus, it is necessary to understand the function of lysine acetylation and succinylation in biological metabolism.

The Kac modification, first found in histones^15^, is involved in the regulation of chromatin structure and gene expression^16^. Concurrent with the development of proteomic approach with high-efficiency, high-throughput, and high-resolution, lysine acetylation has been shown to be widespread and decorates a large range of non-histone proteins^17^. In both prokaryotic and eukaryotic proteins, the acetylation of lysine side chains is a reversible and highly regulated PTM^18^. A broad spectrum of cellular processes, including proteolysis, mRNA processing, autophagy, and proteins contributing to signaling cascades, are related to acetylated non-histone proteins^19^. Ksuc is a newly found reversible PTM and it transfers the succinyl group (100.0186 Da) from the succinyl donor succinyl-CoA to the ε-amino group of a specific lysine residue of the targeted protein, resulting in succinyl-lysine^20^. Succinylation adds a larger structural moiety than acetylation or methylation, which would result in more dramatic structural alteration to lysine and, thus, would likely lead to more significant changes in protein structure and function^20^.

In recent years, protein acetylation modification in three domains of life, such as archaea, bacteria, and eukaryotes, has been widely explored^21^,^22^. However, studies on Kac of plant proteins have lagged behind such studies on mammalian and microbial proteins. In plants, several lysine acetylome studies were implemented in *Arabidopsis thaliana*^18^,^23^, soybean^24^, rice^25^, grape^26^, *Synechocystis* sp^10^, and strawberry^27^, etc. And also a novel database of compendium of protein lysine acetylation (CPLA) had been constructed to meet the desire for complete acetylomes^28^. The study of succinylation in plants has only been conducted in rice^29^ and tomato^30^, to our best knowledge. A comprehensive lysine acetylation and succinylation proteome analysis in *Brachypodium* distachyon L. has not been reported.

With the rapid development of genome-wide analyses in recent years, studies on transcriptomics^31^, proteomics^32^, phosphoproteomics^32^, and glycoproteomics^33^ in Bd21 have been conducted. According to previous studies about the proteomics analysis of lysine acetylation and succinylation, all enzymes in glycolysis, gluconeogenesis, the TCA cycle, and glycogen metabolism were found to be acetylated^13^, and lysine succinylation regulates diverse cellular processes^14^. During the early vegetative stages in Bd21, all the metabolic activity will be rather active. Little is known, however, about protein post-translational acetylation and succinylation during rapid growth stages of Bd21 seedlings. Thus, in this study, we report the first lysine acetylation and succinylation proteome profiling of Bd21 seedling leaves and reveal the central roles of these protein PTMs in the early vegetative stages.

## Results

### Detection of lysine-acetylated and lysine-succinylated peptides and proteins in Bd21 seedling leaves

In this study, we performed global acetylation and succinylation proteome analysis of Bd21 seedling leaves using tryptic digestion, antibody affinity enrichment, and nanoscale liquid chromatography-tandem mass spectroscopy (nano-LC-MS/MS) (Fig. S1). In total, 636 lysine-acetylated sites distributed on 627 peptides in 353 acetylation proteins, nine peptides had two acetylation sites, and 605 lysine-succinylated sites in 262 proteins, were identified with high confidence (Table S1). Detailed information for all identified Kac and Ksuc peptides, and their corresponding proteins, are presented in Table S1. We also deposited mass spectrometry proteomics data in the ProteomeXchange Consortium. The dataset identifiers for the acetylation and succinylation proteomes are PXD003161 and PXD003428, respectively.

**Figure 1:**
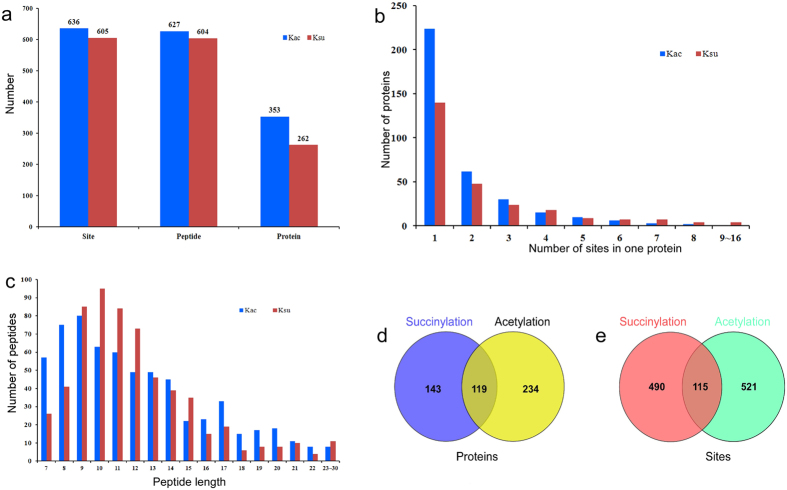
Profile of identified acetylated and succinylated sites, peptides and proteins. **(a)** The statistic analysis of Kac and Ksuc sites, Kac and Ksu peptides and Kac and Ksu proteins. **(b)** Distribution of Kac and Ksuc peptides in one protein. **(c)** Distribution of Kac and Ksu peptides based on their length. **(d)** The statistic analysis of the overlap between the Kac and Ksuc proteins. **(e)** The statistic of the overlap between the Kac and Ksuc sites.

The numbers of acetylated and succinylated proteins, peptides, and sites are shown in Fig. 1a. Among the 353 acetylated proteins, 224 (63%) contained one acetylation site and 62 (18%) contained two acetylation sites (Fig. 1b; Table S2). All identified lysine-acetylated peptides had lengths of 7–28 amino acids (Fig. 1c). Moreover, 605 Ksuc protein sites were distributed on 604 peptides and 262 proteins (Fig. 1a), and 140 (53%) Ksuc proteins had only one lysine-succinylated site (Fig. 1b; Table S2). Four proteins had 10 or more succinylation sites: dihydrolipoyl dehydrogenase 1 (DLD1) (10 sites), heat shock 70 kDa protein (10 sites), succinyl-CoA ligase (12 sites), and ribulose bisphosphate carboxylase large chain (16 sites). All of the identified lysine-succinylated peptides ranged in length from 7 to 30 amino acids (Fig. 1c).

Furthermore, we compared the succinylation proteins and sites with the acetylation dataset, and found that 119 succinylated proteins (Fig. 1d) and 115 succinylated sites (Fig. 1e) were acetylated simultaneously. The 119 proteins included several ATP synthases, two PSI apoproteins, and two DLDs (Table 1). These proteins were associated with energy metabolism, photosynthesis, and protein synthesis, while 115 common sites were derived from 68 common proteins (Table S3). These 68 proteins were included in the 119 proteins and could be regulated simultaneously by succinylation and acetylation.

### Functional annotation and subcellular localization of lysine-acetylated and lysine-succinylated proteins

**Figure 2:**
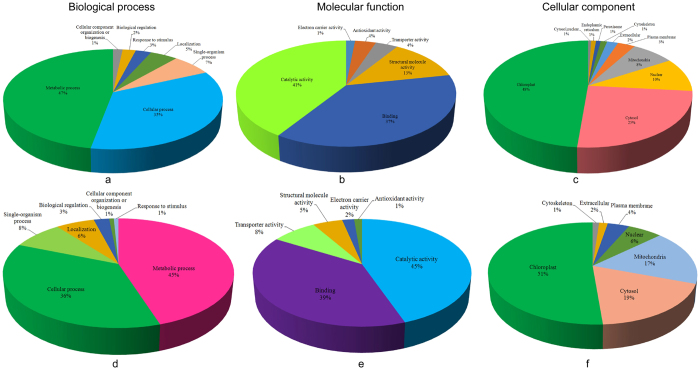
GO classification of the acetylated (**a**–**c**) and succinylated (**d**–**f**) proteins based on biology process, functional distribution and subcellular localization, respectively.

To obtain an overview of the acetylated and succinylated proteins in Bd21 seedling leaves, all identified proteins were subjected to gene ontology (GO) functional analysis, based on their classification into biological processes, molecular functions, and subcellular localization GO categories (Fig. 2; Table S4).

Within the biological process category (Fig. 2a,d), two major classes of acetylated and succinylated proteins were associated with metabolic and cellular processes, accounting for 47 and 35% of the total acetylated proteins, respectively, and 45 and 36% of the succinylated proteins, severally. For the molecular function category (Fig. 2b,e), both of the identified acetylated and succinylated proteins were related to catalytic activity and binding functions, accounting individually for 41 and 37% of all the acetylated proteins, respectively, and 45 and 39% of the succinylated proteins, separately. Regarding the cellular component category (Fig. 2c,f), most of the acetylated proteins were located in the chloroplast (48%), cytosol (25%), and nucleus (10%), and the succinylated proteins were also located in the chloroplast (51%), cytosol (19%), mitochondria (17%), and nucleus (6%), indicating the similar subcellular locations of two types of PTM proteins. Not only the similarities but also differences were found between lysine acetylation and succinylation. According to the functional analysis of GO annotation, we found that the acetylated proteins took part in the structure molecule activity more often than the succinylated proteins. The results from cellular component analysis showed that succinylated proteins were more located at the mitochondria (17%) than nuclear (6%) compared with the acetylated proteins.

### Enrichment analysis of lysine-acetylated and lysine-succinylated proteins

**Figure 3:**
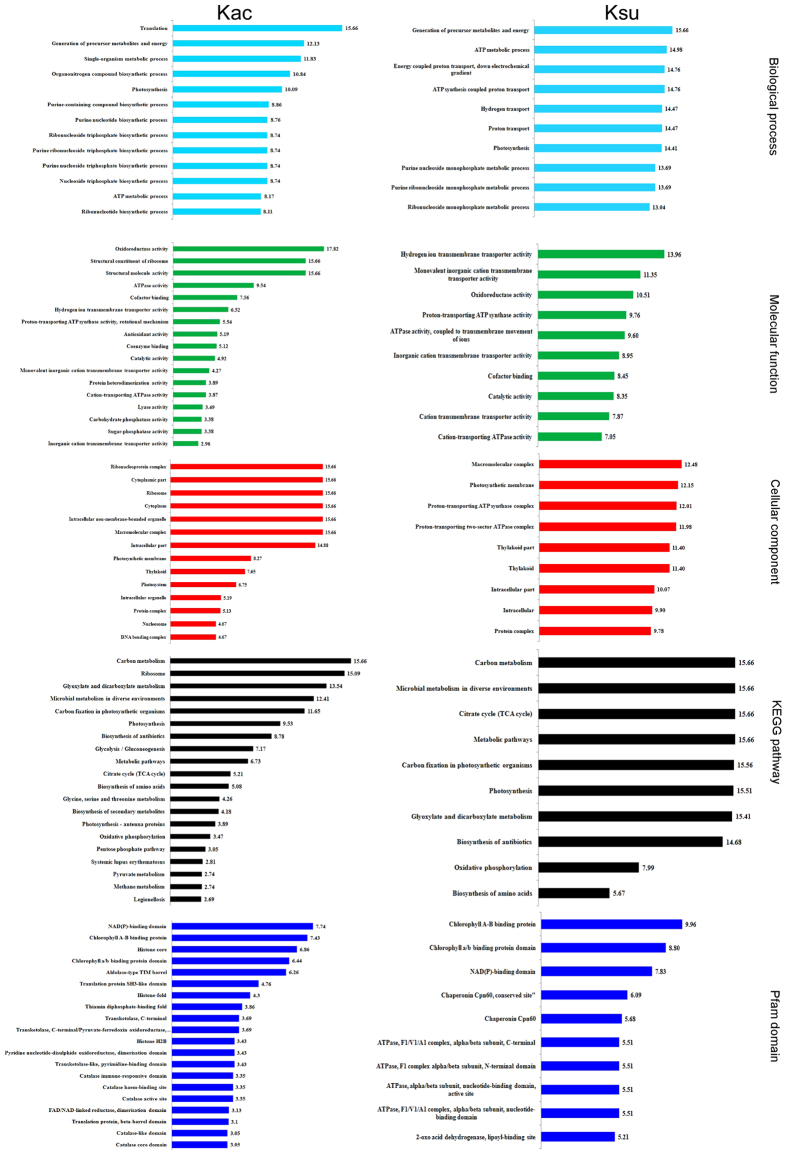
GO-based enrichment analysis of identified acetylated and succinylated proteins. Biology process (watchet bars), subcellular localization (green bars) and molecular function (red bars), KEGG pathway-based enrichment analysis (black bars) and Protein domain enrichment analysis of identified proteins (blue bars) were listed.

To better understand the biological function of the identified proteins, we performed an enrichment analysis of the GO, Kyoto Encyclopedia of Genes and Genomes (KEGG) pathway, and the Pfam domain databases (Fig. 3; Table S5). Gene ontology enrichment analysis based on the biological process category (wathet bar) showed that translation, photosynthesis, and ATPase activity were enriched in acetylated proteins (Fig. 3; Table S5-1). Similarly, these processes were enriched significantly in succinylated proteins (Table S5-2). Based on the enrichment results of the molecular function category (Fig. 3, green bars), oxidoreductase activity, structural constituents of ribosomes, and ATPase activity were enriched primarily in the acetylated proteins (Table S5-3). However, in the succinylated proteins, hydrogen ion transmembrane transporter activity ranked first, followed by transmembrane transporter activity (Table S5-4). There were a few differences between these two modifications. When enrichment analysis of the cellular components was performed, the acetylated-proteins were enriched significantly in the ribonucleoprotein complex, ribosome, and cytoplasm (Fig. 3, red bars; Table S5-5). These succinylated proteins were enriched significantly on the macromolecular complex, photosynthetic membrane, and proton-transporting ATP synthase complex (Table S5-6).

The KEGG pathways of carbon metabolism, photosynthesis, and carbon fixation in photosynthetic organisms were enriched significantly in the acetylated proteins (Fig. 3, black bars; Table S5-7). Additional acetylated proteins were mapped to KEGG pathways, including oxidative phosphorylation, biosynthesis of amino acids, the TCA cycle, and glycolysis/gluconeogenesis. The KEGG pathway enrichment results also showed that carbon metabolism, the TCA cycle, carbon fixation, and photosynthesis were enriched significantly in the succinylated proteins (Fig. 3, black bars; Table S5-8). Furthermore, Pfam domains, including the NAD(P)-binding domain and the chlorophyll a/b binding protein domain, were enriched significantly in both acetylated and succinylated proteins (Fig. 3, blue bars; Tables S5-9,5–10).

### Motifs and secondary structures of lysine-acetylated and lysine-succinylated peptides

Previous studies of both eukaryotic and prokaryotic cells identified the preferences for amino acid residues at particular positions surrounding acetylated/succinylated lysines. Thus, to further evaluate the nature of the acetylated and succinylated lysines in Bd21, we investigated the sequence motifs in all identified lysine residues, using the Motif-X program.

**Figure 4:**
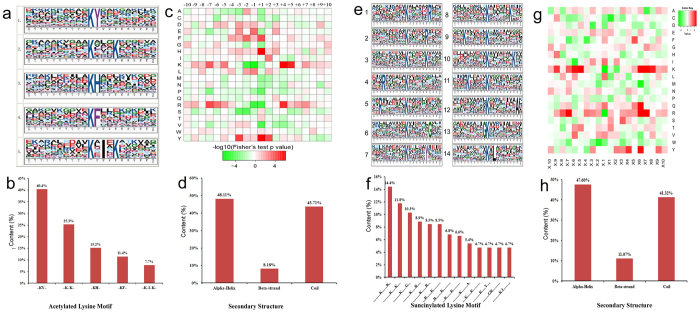
Properties of the identified acetylated and succinylated peptides. Enrichment analysis of acetylated proteins motifs **(a)** and succinylated proteins motifs **(e)**. The height of each letter corresponds to the frequency of that amino acid residue in that position. The central K refers to the acetylated/succinylated lysine. Motif-X software was used to do motif analysis. Content of these identified peptides containing acetylated **(b)** succinylated lysine **(f)** in each motif. Heat map of the amino acid compositions of the acetylation **(c)** and succinylation **(g)** sites showing the frequency of the different types of amino acids around the succinylated lysine. The content of secondary structures (α-helix, β-strand, coil) in each identified acetylated **(d)** and succinylated **(h)** protein.

A total of five conserved motifs, with amino acid sequences from −10 to +10 surrounding the acetylated lysine, were extracted from 636 acetylated peptides (Fig. 4a; Table S6-1). These motifs included -K(ac)Y-, -K(ac)---K-, -K(ac)H-, -K(ac)F-, and –K(ac)-I-K- (Fig. 4a). Two distinct types of residues were located upstream of the acetylated lysine: a positively charged residue including histidine (H) or lysine (K), and a residue with aromatic groups such as tyrosine (Y) and phenylalanine (F). KY was the most common combination, represented by 120 (40%) of the enrichment motifs (Fig. 4b).

Similarly, 14 conserved motifs were identified in succinylated proteins (Fig. 4e; Table S6-2). However, the same motifs were not in the two PTMs. Two distinct types of residues were located upstream/downstream of the succinylated lysine: a positively charged residue including K or arginine (R), and a residue without charge, such as alanine (A), glycine (G) or leucine (L). -K(suc)-----K- was the most common combination, represented by 57 (12%) of the enrichment motifs (Fig. 4f).

To further analyze these motifs, we performed hierarchical cluster analysis (Fig. 4c,g). Based on the position of the residues and other properties of the residues surrounding the acetylated lysine, these motifs could be classified into two categories: the +4 or +5 positions, which were alkaline residues with long side chains (K or R), and the +1 or +2 positions, which were residues with long side chains (Y, H, or F; Fig. 4c). These succinylated lysines also could be classified into two categories: the +6, +7, or +8 positions or the −6 or −7 positions, which were alkaline residues with long side chains (K or R), and the +4 or +6 positions, which were residues with long side chains (Y; Fig. 4g). These new motifs in Bd21 would potentially provide an acetylation/succinylation binding loci for future studies.

We conducted the Motif analysis on these 115 common sites (Table S3), and found that most of these sites were belonged to -K------G---, ---R------K, ---K------K- etc. These motifs indicated that around the lysine residues, some alkaline residues with long side chains (K or R) were more easily to be acetylated and succinylated. This phenomenon is consistent with above analysis.

We used the algorithm NetSurfP to predict the secondary structures of these acetylated and succinylated proteins (Fig. 4d,h). The results indicated that the α-helix, coil, and β-strand structures accounted for 48, 44, and 8% of all the acetylated sites, respectively (Table S7-1), and 48, 41, and 11% of 605 succinylated sites, separately (Table S7-2). These results identified two modification proteins with similar structural characteristics. Furthermore, we also checked the secondary structure of these 115 common sites, found that the coil with the possibility of 50.4%, α-helix with the possibility of 35.7% and the β-strand with the possibility of only 13.9%. This indicates that the PTMs tend to occur at the loose area (coil).

### Conservation analysis of the acetylated and succinylated proteins

**Figure 5:**
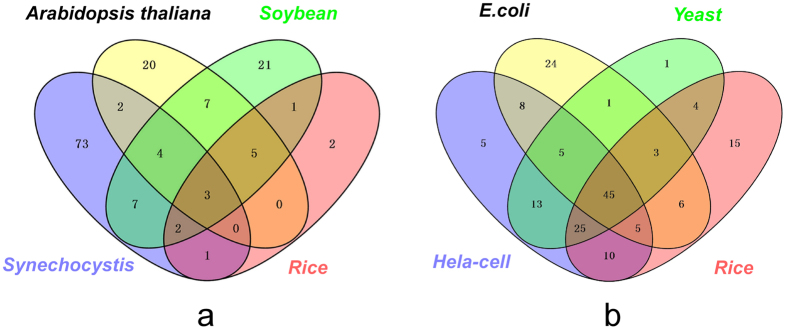
Conservation analysis of the acetylated (**a**) and succinylated (**b**) proteins in seedling leaves of Bd21. (**a**) All the identified acetylated proteins in Bd21 compared with *Oryza sativa*, *Arabidopsis thaliana*, *Synechocystis* sp. PCC 6803, and *Glycine max*. **(b)** All the identified succinylated proteins compared with rice, *E. coli*, *S. cerevisiae* and *H. sapiens*.

To analyze conservation of acetylated/succinylated protein function between Bd21 and other species, we used the sequences of the identified proteins to perform a BLAST search and estimated the degree of conservation of acetylated proteins among rice, *A*. *thaliana*, *Synechocystis* sp. PCC 6803, and soybean (Fig. 5a). We also used the succinylated sequences to perform a BLAST search among *Oryza sativa* L., *E. coli*, *S. cerevisiae*, and *H. sapiens* (Fig. 5b). The parameters were set as follows: E-value <1 × 10^−10^, score ≥80, and identity ≥30%.

The results showed that 148 (42%) of the identified acetylated proteins in Bd21 had orthologous proteins in the other four organisms. Among the 353 identified acetylated proteins in Bd21, only 14 had highly conserved orthologs with an average identity of 69% in the model monocot plant species rice (Table S8-1). However, 41 had conserved orthologs with an average identity of 72% in the model dicot plant species *A. thaliana*. We also compared the acetylated proteins with those in soybean and found that 50 showed conservation with Bd21, with an average identity of 69%. Furthermore, we compared the identified acetylated proteins with those from *Synechocystis* sp. PCC 6803. We found 92 proteins that were orthologous with Bd21 proteins, but the average identity was only 53%, probably due to their distant genetic backgrounds. Further analysis demonstrated that three glyceraldehyde-3-phosphate dehydrogenases (GAPDH), gi357114230, gi357144527, and gi357163943, existed in these five species, indicating their crucial roles and conservation in these organisms.

A total of 170 (65%) of the homologous succinylated proteins in Bd21 were also identified in the other four species. According to our analysis, 45 of the 262 succinylated proteins were found in all five species (Fig. 5b; Table S8-2). These proteins included ATP synthase, GAPDH, and DLD, while 113 proteins had highly conserved orthologs in the rice (Table S8-2). In addition, 97 succinylated proteins were in both *E. coli* and *S. cerevisiae*. Most of these proteins participated in the TCA cycle and protein/amino acids synthesis metabolism. This also indicated that succinylation is conserved in different species and plays important roles in energy and protein metabolism.

We also conducted the comparison with the data from *Arabidopsis thaliana* and rice to see how many of the identified acetylated sites could also be succinylated. Based on our results, we found that when compared with rice, eight sites had both PTMs. Comparison with the acetylated database from *Arabidopsis thaliana* showed that only four acetylated sites could also be succinylated (Table S10?).

### Screening of significant acetylated and succinylated proteins in Bd21 seedling leaves

To further study these identified proteins and determine the locations of Kac and Ksuc sites in the conserved proteins, we aligned them with previously identified acetylated/succinylated proteins and predicted their three-dimensional (3D) structures (Figs S2 and S3). Two DLDs that play a significant role in metabolism and energy production were found to be both acetylated and succinylated. Ten succinylation sites were identified in the DLD1 (Fig. S2a). Four sites (K-140, K-195, K-405, and K-425) were also found to be acetylated (Fig. S2a; Table S3).

To further study the conservation and structural characteristics of DLD1 (gi357132047), we performed sequence alignment (Fig. S2a) and predicted the 3D structure (Fig. S2b). Both K-140 and K-405 were somewhat conserved in these four species. The 3D structures showed that the acetylated and succinylated sites were more likely to be located in the α-helix and coil structures. This also verified our secondary structure prediction (Table S7-1,S7-2).

According to conservation analysis, three GAPDHs were found to be both acetylated and succinylated in Bd21 (Fig. S3). In the protein gi357114230, we identified two sites, K-126 and K-135, with both acetylation and succinylation. The alignment results indicated that site 126 was highly conserved among the four species. Sites 126 and 135 were located at the coil (Table S7-1,S7-2). For the protein gi357144527, only one site (K-217) was identified at α-helix with both PTMs. In protein gi357163943, four sites (K-177, 235, 292, 319) were identified to be both acetylated and succinylated at α-helix or coil. This was also confirmed by our previous prediction with the algorithm NetSurfP (Table S7-1,S7-2). Our findings provide valuable information for the future study of acetylation and succinylation of these proteins.

### Protein-protein interaction analysis of acetylated and succinylated proteins

We conducted protein-protein interaction (PPI) analysis using Cytoscape software to further understand the cellular processes regulated by acetylation and succinylation. The PPI network of acetylation modification had 210 acetylated proteins as nodes connected by 7094 identified direct physical interactions, and 148 succinylated proteins as nodes with a combined score higher than 0.70 as obtained from the STRING database (Fig. S4; Table S11). This analysis would promote detecting the hypothetical functions of the identified proteins.

Based on the protein function and cellular processes, nine categories of acetylated proteins and eight groups of succinylated proteins are shown in Fig. S4. Sixty-one of the acetylated proteins were involved in the translation, ribosomal structure, and biogenesis group. This group was the largest in the Kac PPI network, indicating that these acetylated proteins played vital roles in protein translation. Thirty-one of the proteins were in the energy production and conversion group, constituting the second largest group. They interacted closely with the amino acid and carbohydrate transport and metabolism, post-translational modification, and chaperones group.

For succinylated proteins, the largest cluster, with 44 (30%) succinylated proteins, comprised the energy production and conversion metabolism group. The second largest cluster, with 25 succinylated proteins, belonged to the translation/ribosomal structure/biogenesis group, which differed from acetylated proteins. Carbohydrate and amino acid metabolism were also important in our PPI network, and had intimate relationships with the energy production and conversion metabolism group. Two proteins, named ubiquitin-like proteins and 14-3-3-A, were connected closely with other proteins (Fig. S4). These results provided a possible PPI network resource for future studies of acetylated and succinylated proteins.

### Western blot verification of protein acetylation and succinylation during Bd21 seedling growth

**Figure 6:**
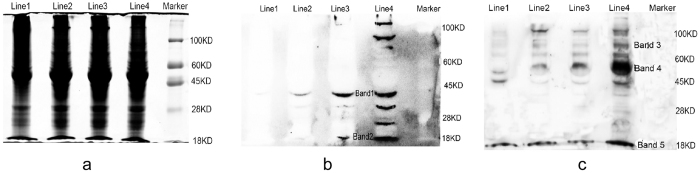
Western blotting validation of the succinylated proteins in the leaves of Bd21 with the Pan-acetylated and Pan-succinylated antibody. (**a**) The SDS-PAGE of the proteins in four periods leaves. (**b**) The Western blotting signal of the acetylated proteins. (**c**) The Western blotting signal of the succinylated proteins. Line 1, Line 2, Line 3 and Line 4 are the one, two, three and four leaf period, respectively. Marker contains 100KD, 60KD, 45KD, 28KD and 18KD. Band 1, 2, 3, 4 and 5 were also analyzed by the LC-MS/MS.

We used western blotting with the pan anti-acetyllysine and succinyllysine antibody, combined with LC-MS/MS analysis, to validate the proteome results and understand the protein acetylation and succinylation changes during Bd21 seedling growth (Fig. 6; Table S12). We photographed seedling leaves and determined their physiology indices to confirm rapid growth of Bd21 during the vegetative period (Fig. S5). Plant height, leaf length, and root length increased rapidly during seedling development. Water content also increased significantly during this period. With the pan antibody, we detected variation of acetylation and succinylation in four-leaf periods (Fig. 6). As the seedling leaves grew, the protein acetylation and succinylation levels increased. Several bands with strong signals were detected by western blot analysis, with multiple properly controlled biological and technical replicates (Fig. 6b,c). Then, these bands were subjected to LC-MS/MS analysis and several proteins were identified, including ATP synthase subunit alpha/beta, ribosomal proteins, elongation factor Tu, and histones (Table S12). Most of the identified proteins were related to photosynthesis, energy metabolism, and protein synthesis. These proteins were also identified in our proteome analysis (Table S1), indicating the reliability of our acetylome and succinylome dataset.

## Discussion

In this study, we identified 636 lysine acetylation sites in 353 proteins, and 605 succinylation sites in 262 proteins in seedling leaves of Bd21, which constituted a large dataset. We also compared our acetylation dataset with previous studies, and 164 acetylated proteins were not found homologous proteins in the *Synechocystis sp*. PCC 6803, *Arabidopsis thaliana*, soybean, rice and strawberry (Table S9). However, the number of acetylated and succinylated proteins, and peptides, was much lower than the number of phosphorylated proteins and peptides in Bd21 seedling leaves^32^ and bread wheat^34^. Nevertheless, we still considered that protein acetylation played a major role in metabolic regulation, which is comparable to phosphorylation^13^.

Ksuc is a newly found reversible PTM existing in both eukaryotes and prokaryotes^20^. Previous studies on Ksuc focused mainly on eukaryotes such as HeLa cells^14^, yeast and mouse liver mitochondria^35^,^36^, as well as a few prokaryotes such as *Mycobacterium tuberculosis*^37^,^38^ and *E. coli*^39^. However, to our knowledge, only two succinylated proteome studies in plants (rice and tomato) have been reported. The number of our acetylated and succinylated sites and proteins was similar to those for rice (699 acetylated sites in 389 proteins and 665 succinylated sites in 261 proteins). Compared with rice and tomato^30^ (347 sites of lysine succinylation in 202 proteins), 85 proteins were not identified of homologous proteins in previous studies (Table S9). Thus, our dataset is a much larger resource for the future studies of plant development mechanisms.

In this study, two new acetylated motifs (K***K and K*I*K) in plants were identified for the first time in Bd21, compared with previous studies in *Synechocystis* sp. PCC 6803^10^, grape^26^, and strawberry^27^. This provides potential acetylation binding loci for future studies in plants. The KY motif was highly conserved relative to grape^26^ and strawberry^27^. Similarly, 12 new succinylated motifs were identified in Bd21 for the first time, compared with previous plant succinylation proteome studies. Two motifs, -K(su)------R- and ---K------K(su)-, were also identified in tomato^30^. However, based on our data, we did not identify the same motifs as from previous studies in rice^29^. These 12 new motifs in plants would provide possible succinylation binding loci for future studies.

From our Western blotting analysis (Fig. 6), we concluded that the acetylation and succinylation levels increased steadily along with the development of Bd21 seedling leaves. According to Zhao *et al*.^13^, all enzymes in glycolysis, gluconeogenesis, the tricarboxylic acid (TCA) cycle, and glycogen metabolism were found to be acetylated, demonstrating the important roles of Kac in various metabolic pathways. Ksuc is a widespread and important PTM in both eukaryotes and prokaryotes, and regulates diverse cellular processes^14^. Thus, we concluded that the increased levels of acetylation and succinylation could play important roles in plant development during the rapid growth period.

Carbon is metabolized continuously in plants and is essential for energy circulation and plant survival. Three respiratory pathways, including glycolysis, the mitochondrial electron transport chain, and the TCA cycle, are essential for energy supply to numerous cellular functions^40^. The pyruvate dehydrogenase complex is a complex of three enzymes [pyruvate dehydrogenase (PDH), dihydrolipoyl transacetylase (DLAT), and DLD], which catalyzes the overall conversion of pyruvate to acetyl-CoA and CO_2_. In our study, we identified four acetylated and six succinylated proteins in this complex (Table S1). Among these proteins, four sites were found to be both acetylated and succinylated in protein DLD1 (gi357132047), this proteins also with ten succinylated sites, as shown in Fig. S2a. Both modifications were found more frequently at α-helix and coil structures (Fig. S2b). This complex was also found in rice with 6 and 5 PyDC subunits being modified by Kac and Ksuc, respectively. The intensive succinylation of the acetyl-CoA metabolism-related enzymes indicated complex interactions between two PTMs^29^. Thus, we speculated that the activity of the complex could be regulated by both acetylated and succinylated modifications.

In the TCA cycle, malate dehydrogenase1 (MDH1) is a homodimeric enzyme which catalyzes the reversible oxidation of malate to oxaloacetate, in the presence of NAD, and, thus, plays a major role in central metabolism^41^. This protein was identified to be both acetylated and succinylated in our study (Table 1). A previous study showed that the acetylation of MDH1 enhanced its enzymatic activity dramatically and, subsequently, increased the intracellular levels of NADPH to promote adipogenic differentiation in preadipocyte cells^42^. This protein was also found to be acetylated in *Arabidopsis*^18^. These lysine-acetylated residues are located at the dimer-dimer interface of two subunits in the tetrameric enzyme MDH. The two lysine residues are important for the oligomeric integrity of the protein complex in *Chloroflexus aurantiacus*^43^ and *Arabidopsis*^18^. They are also important for the network of electrostatic interactions. In addition, five sites (K-118, 181, 245, 248, and 333) were found to be succinylated in this protein, but there was no overlap with acetylated sites. This enzyme was also identified to be succinylated in tomato^30^. It is likely that both PTMs participate simultaneously in regulating the activity of this enzyme.

In glycolysis, GAPDH catalyzes the conversion of D-glyceraldehyde 3-phosphate (G3P) to 3-phospho-D-glyceroyl phosphate. Plastid GAPDH forms PPI with other glycolytic enzymes or Calvin cycle enzymes in the chloroplast^44^. Increased GAPDH acetylation leads to increased activity in glycolysis and decreased activity in gluconeogenesis, thus revealing that acetylation of GAPDH controls carbon flux, in favor of glycolysis in cells grown on glucose in bacteria^45^. Three GAPDH proteins (1, A, and B) were found to be both acetylated and succinylated in our study (Fig. S3). Two sites (K-126 and 135), one site (K-217), and four sites (K-177, 235, 292 and 319) were identified with both PTMs at α-helix or coil regions in proteins gi357114230, gi357144527, and gi357163943, respectively. Acetylated Lys residues might have an impact on PPI and are completely exposed on the protein surface, protruding like needles that remove a surface charge^18^. Although the function of succinylation on GAPDH is still not clear, we could predict that both PTMs might function simultaneously to regulate carbon flux.

Along with reactivation of the glycolysis and TCA cycles, the mitochondrial electron transport/ATP synthesis metabolism was restored. The ATP synthase (F_0_F_1_-ATP synthase) is one of the most beautiful as well as one of the most unusual and important^46^. ATP synthase carries out the most prevalent chemical reaction in the biological world, and it is one of the most ubiquitous, abundant proteins on Earth^47^. In this study, 13 ATP synthase subunits were identified to be succinylated and eight of them were also found to be acetylated, including α, β, ε, γ, b, d, and o (Table 1). Not only succinylation and acetylation, but other PTMs such as oxidative phosphorylation or glutathionylation, and nitrosylation, were also found on ATP synthase^48^. The ATPeF0D, ATPeF1A, ATPeF1B, and ATPeF1G of ATP synthase subunits were identified to be both acetylated and succinylated in rice seeds^29^. It would be interesting to investigate the crosstalk among the different PTMs in the regulation of ATP synthase. These results suggested that Kac and Ksuc might cooperate or compete with each other on the same protein.

**Figure 7:**
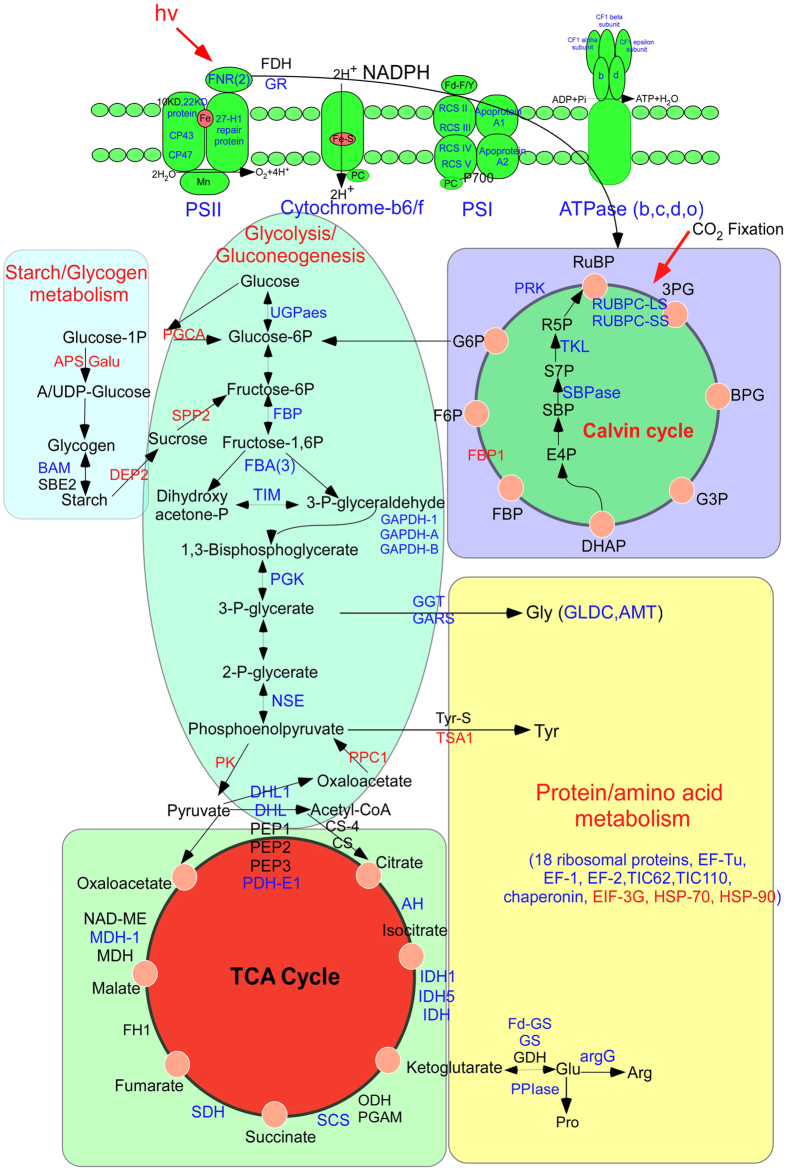
The pathway of lysine acetylation and succinylation proteins involved in photosynthesis and energy metabolism, protein/amino acids metabolism. The proteins both to be succinylated and acetylated were marked with blue colour. The succinylated proteins were marked in black colour and the acetylated proteins were marked with red colour. UGPase: UTP--glucose-1-phosphate uridylyltransferase, TIM: triosephosphate isomerase, TKL: transketolase, SCS: succinyl-CoA ligase, SDH: succinate dehydrogenase, RPE: ribulose-phosphate 3-epimerase, PDH-E1: pyruvate dehydrogenase E1 component subunit alpha-1, AH: aconitate hydratase, PGK: phosphoglycerate kinase, NADME: NAD-dependent malic enzyme 59 kDa isoforms, MDH: malate dehydrogenase, IDH: isocitrate dehydrogenase [NADP], GAPDH: glyceraldehyde-3-phosphate dehydrogenase, FH: fumarate hydratase 1, FBA: fructose-bisphosphate aldolase, FBP: fructose-1,6-bisphosphatase, NSE: enolase, PEP: dihydrolipoyllysine-residue acetyltransferase component, DHL: dihydrolipoyl dehydrogenase, CS: citrate synthase, BAM: beta-amylase, ODH: 2-oxoglutarate dehydrogenase, PGAM: 2,3-bisphosphoglycerate-independent phosphoglycerate mutase, SBE2: 1,4-alpha-glucan-branching enzyme 2, Tyr-s: tyrosine--tRNA ligase, GGT: glutamate--glyoxylate aminotransferase 2, Fd-GS: ferredoxin-dependent glutamate synthase, GLDC: glycine dehydrogenase, PPIase: peptidyl-prolyl cis-trans isomerase, AMT: aminomethyltransferase, GS: glutamine synthetase, GDH: glutamate dehydrogenase, ARGG: argininosuccinate synthase, EIF: eukaryotic initiation factor, EF: elongation factor, FD: formate dehydrogenase, GR: glutathione reductase, RCS: reaction center subunit, PRK: phosphoribulokinase, RUBPC: ribulose bisphosphate carboxylase, SBPase: sedoheptulose-1,7-bisphosphatase, TKL: transketolase, FNR: ferredoxin--NADP reductase, GALU: UTP--glucose-1-phosphate uridylyltransferase, APS: glucose-1-phosphate adenylyltransferase small subunit, DPE2: 4-alpha-glucanotransferase DPE2, PPC1: phosphoenolpyruvate carboxylase 1, PK: pyruvate kinase, PGCA: phosphoglucomutase, SPP2: sucrose-phosphatase 2, EIF-3G: eukaryotic translation initiation factor 3 subunit G, HSP70/90: heat shock cognate 70 kDa/90 kDa, TSA1: tryptophan synthase beta chain 1.

Photosynthesis converts the energy of sunlight quanta into chemical bonds in which it is stored. In this study, we identified 60 acetylated and 52 succinylated proteins that were involved in the photosynthetic pathway (Table S1; Fig. 7). In particular, 28 of these proteins were found to be both acetylated and succinylated (Table 1). Concurrent with light harvesting, NADPH is generated by electron flow. In the Calvin-Benson cycle, ATP is synthesized by ATP synthase to fix carbon dioxide by utilizing the proton motive force^49^. Four proteins in the Calvin cycle are involved in the regeneration of ribulose-l, 5-bisphosphate (RubP), including ribulose-l,5-bisphosphate carboxylase/oxygenase (Rubisco), phosphoribulokinase (PRK), sedoheptulose-l,7-bisphosphatase (SBPase), and the chloroplastic isoform of fructose-1,6-bisphosphatase (cp-FBPase)^50^. These proteins and transketolase (TK) were found in our study to be both acetylated and succinylated (Fig. 7). As we know, Rubisco catalyzes the limiting step of photosynthetic capacity^51^ and accounts for a large fraction of leaf nitrogen^52^. According to previous reports, Rubisco is the most abundantly lysine-acetylated, with nine different lysine-acetylated sites identified in the chloroplast-encoded large subunit^18^. Sixteen succinylated sites were identified in this protein (Table S1-2). The function of trimethylation at Lys-14 is considered to play a role in the regulation of the protein-protein interactions of Rubisco with other proteins^53^. Similarly, the same could be suggested for lysine acetylation^18^, because lysine acetylation functions as a binding motif for bromodomain-containing proteins^12^. The treatment of leaf extracts with hSIRT3 deacetylase caused a 40% increase in maximum catalytic activity of Rubisco^18^; furthermore, this would affect photosynthesis. In our study, 10 sites were found to be both acetylated and succinylated in seedling leaves of Bd21, which might affect the rate of photosynthesis. Among the 10 sites, six sites (14, 146, 175, 252, 356, and 463) were also identified in *A*. *thaliana*, indicating the conservation of Rubisco acetylation in different species. Our results provide useful information for future studies of the effects of acetylation/succinylation on photosynthesis and carbon dioxide fixation.

Apart from these referred similarities of these proteins with two PTMs, we also reanalyzed the dataset to find their differences. These histones were found with acetylation modification in H2A, H2B, H4 and H1, indicating that the Kac had higher tendency to occur in histones than Ksu. This also verified previous studies that acetylation is rather conserved in histone. For the pyruvate dehydrogenase complex, the acetylation tends to occur in the E1, while succinylation had no specific tendency on this complex, no matter E1, E2 and E3 according to our study. This also showed that these two PTMs regulate the activity of the complex synergistically. Apart from that, we also found that peroxidase was found with only acetylation modification. We didn’t find this kind of proteins with succinylation modification in the present study. We speculated that the acetylation could regulate the activity of peroxidase to response to adverse stress during plant development. Based on above results, we concluded that some specific functions were regulated by acetylation or succinylation during rapid development of Bd21 seedling.

All the proteins identified from our large-scale analysis were involved in numerous metabolic activities (Fig. 7). Kac and Ksuc could play a role as a switch, to control some key enzyme activities and assure the proper function of Bd21 development. In this study, we provide the first details of crosstalk between the reversible Kac and Ksuc in Bd21, which would supply useful information to detect the mechanism of both PTMs in regulating the rapid development of seedling leaves in Bd21.

## Materials and Methods

### Plant Materials and Growth Conditions

We used Bd21 as plant material in this study. We processed the seeds, cultivated the seedlings and the seedling growth stages were determined according to Zhang *et al*.^33^. The seedling leaves with three biological replicates in the three-leaf stage were sampled and frozen at −80 °C for subsequent protein extraction.

### Preparation of Proteins and In-solution Digestion

Total proteins from the leaves of Bd21 seedlings were extracted according to the procedure of Lv *et al*.^32^, with minor modifications. About 500 mg of fresh leaves were grinded by liquid nitrogen firstly, then the cell powder was transferred to 5 mL centrifuge tube and sonicated three times for 1 minute each time on ice using a ultrasonic processor (Scientz) in lysis buffer (8 M urea, 1% Triton-100, 65 mM DTT and 1% Protease Inhibitor Cocktail, 3 μM TSA, 50 mM NAM). After that, the remaining debris was removed by centrifugation at 12,000 g at 4 °C for 10 min. Finally, the protein was precipitated with cold 15% TCA overnight at −20 °C. After centrifugation at 12,000 g at 4 °C for 15 min, the supernatant was discarded. The remaining pellets were washed with cold acetone with 50 mM DTT, 1 mM PMSF for three times. These pellets were redissolved in buffer (8 M urea, 100 mM TEAB, pH 8.0) and the concentrations were determined with 2-D Quant kit (GE Healthcare, USA) according to the manufacturer’s instructions. The protein solutions were stored at −80 °C for later use.

For digestion, firstly, we used 10 mM DTT to reduce the protein solution for 1 h at 37 °C, and then, alkylated for 45 min at room temperature with 20 mM IAA that in darkness. Secondly, for trypsin digestion, the protein samples were diluted by adding 100 mM NH_4_CO_3_ to urea concentration less than 2 M. Finally, for the first digestion, trypsin was added at 1:50 trypsin-to-protein mass ratio overnight, and 1:100 trypsin-to-protein mass ratio for a second 4 h digestion, respectively.

### Immunoaffinity Enrichment of Lysine Acetylated and Succinylated Peptides

To enrich Kac and Ksu peptides, tryptic peptides were dissolved in NETN buffer (100 mM NaCl, 1 mM EDTA, 50 mM Tris-HCl, 0.5% NP-40, pH 8.0). Then, these mixtures were incubated with pre-washed antibody beads at 4 °C overnight with gentle shaking at the same time (PTM Biolabs). The details were followed the methods of Fang *et al*.^27^ and He *et al*.^29^. Finally, these peptides were cleaned with C_18_ Zip Tips (Millipore) with the help of manufacturer’s instructions.

### LC-MS/MS Analysis

Peptides were dissolved in 0.1% FA and then loaded onto a reversed-phase pre-column (Acclaim PepMap 100, Thermo Scientific). A reversed-phase analytical column was used to perform peptide separation (Acclaim PepMap RSLC, 50 μm, Thermo Scientific). The gradient was comprised of an increase from 6% to 22% solvent B (0.1% FA in 98% acetonitrile) for 26 min, 22% to 35% for 10 min and climbing to 80% in 3 min, and then holding at 80% for the last 3 min. These processes are at a constant flow rate of 280 nl/min on an EASY-nLC 1000 UPLC system. These peptides were analyzed by Q Exactive^TM^ Plus hybrid quadrupole-Orbitrap mass spectrometer (Thermo Fisher Scientific) in the following steps.

These peptides were subjected to NSI source and then followed by tandem mass spectrometry (MS/MS) in Q Exactive^TM^ Plus (Thermo), at the same time, coupled online to the ultra performance liquid chromatography (UPLC) instrument, mass window for precursor ion selection is 2.0 m/z, charge state with >5 as exclusion parameters. Peptides were selected for MS/MS using normalized collisional energy (NCE) setting as 30; ion fragments were detected in the orbitrap at a resolution of 17,500. Simultaneously, intact peptides were detected in the orbitrap at a 70,000 resolution. A data-dependent procedure that alternated between one MS scan followed by 20 MS/MS scans was applied for the top 20 precursor ions above a threshold ion count of 1.5E4, in the MS survey scan with 30.0 s dynamic exclusion. The electrospray voltage was 2.0 kV. Automatic gain control (AGC) was used to prevent overfilling of the ion trap. For MS scans, the m/z scan range was 350 to 1800 Da. 5E4 ions were accumulated for generation of MS/MS spectra.

### Database Searching

MaxQuant with integrated Andromeda search engine (v.1.4.1.2) was used to process the resulting MS/MS data. Tandem mass spectra were searched against *Brachypodium distachyon* protein database (34,310 entries) in Phytozome (http://www.phytozome.net/search.php; version 10.2), concatenated with reverse decoy database. To increase the accuracy of identified acetylated proteins, the limiting conditions were as follows: Trypsin/P (Promega) was specified as cleavage enzyme, which will allow up to 5 modifications per peptide, 4 missing cleavages and 5 charges. Mass error for precursor ions was set to 5 ppm and 0.02 Da for fragment ions. Acetylation/succinylation on Lys and acetylation/succinylation on protein N-terminal were specified as variable modifications. Carbamido methylation on Cys was specified as fixed modification. Oxidation on Met was specified as fixed modification. False discovery rate (FDR) thresholds were specified at 1% for protein, peptide and modification site. 7 was set as the minimum peptide length. 0.75 was set as the site localization probability. The other parameters were set to default values in MaxQuant analysis.

### Bioinformatics Analysis of Lysine Acetylated and Succinylated Peptides and Proteins

Gene ontology (GO) annotation proteome was derived according to He *et al*.^29^. All these identified proteins were classified by GO annotation based on three categories: cellular component, biological process and molecular function. After that, the KEGG pathway was annotated using online service tool KEGG Automatic Annotation Server (KAAS), and the annotation results were mapped using the online service tool KEGG Mapper. For subcellular localization predication, WoLF PSORT (http://wolfpsort.org/), the UniprotKB (http://www.uniprot.org/) database and CELLO (http://cello.life.nctu.edu.tw/) were used to predict the subcellular localization^54^,^55^. The most likely location of a given protein was determined by similar results from at least two programs. Data sets of the protein family motif (Pfam) were used to identify domains according to Finn *et al*. (http://pfam.sanger.ac.uk/)^56^. Fisher’s exact test (two-tailed test) was used to test for GO/KEGG pathway/Pfam domain enrichment analysis. Correction for multiple hypothesis testing was carried out using standard FDR control methods. The GO/pathway/domains with a corrected *p*-value < 0.05 was considered significant. Amino acid sequence motifs were analyzed using motif-X (http://motif-x.med. harvard.edu/)^57^. Peptide sequence which cuts out 10 amino acids upstream and downstream of identified acetylation/succinylation site from identified protein sequence was used as foreground sequence for motif analysis. And all the database protein sequences were used as background. The other analysis parameters: modified acid amino “central character” set as ‘K’ (lysine), foreground peptides sequence length “width” set at 21, minimal number of peptide occur in one motif “occurrences” set at 20, motif analysis statistics test significance threshold value set at 0.0000001. Enrichment-based clustering analysis of these motifs was done according to Fang *et al*.^27^. The “heatmap. 2” function from the “gplots” R-package was used to do the heat map cluster analysis. eggNOG (http://eggnog.embl.de/version_3.0/) was used to obtain the EuKaryotic Orthologous Groups (KOG) numbers of those acetylated/succinylated proteins. After that, the Search Tool for Retrieval of Interacting Genes/Proteins (STRING) database (http://string-db.org/) was used for PPI analysis with these KOG numbers. The standard is that had a high confidence score of >0.7, based on coexpression and experiment conditions. Cytoscape (version 3.0) software was used to display the network^58^. Secondary structures were predicted using NetSurfP. We used the Phyre2, engine v 2.0 (http://www.sbg.bio.ic.ac.uk/ phyre2/html/page.cgi? id=index) to predict the 3D structure of these acetylated and succinylated proteins. Further, BLASTP was conducted to evaluate their conservation across species according to previous report^34^.

### Western Blotting

We used 5% skim milk powder to block the membranes in phosphate-buffered saline that containing 0.1% Tween-20 for 1 h at room temperature. After that, the membrane was incubated with the specific antibody anti-acetyl and anti-succinyl lysine antibody (1:1000, in TBS/5% skim milk powder) (PTM Biolabs, Hangzhou) overnight at 4 °C. Second day, we washed the membrane three times with TBST buffer (25 mM Tris-HCl, pH 8.0, 150 mM NaCl, 0.1% Tween 20). Last, the membrane was incubated with horseradish peroxidase-conjugated goat antirabbit/antimouse antibody for 1 h at 37 °C (1:2500 dilutions). After washed with TBST buffer, it was visualized with enhanced chemiluminescence (ECL) immunoblotting detection reagents (GE Healthcare, USA). The density of each band was determined with a fluorescence scanner (Image Quant TL, GE Healthcare, USA).

## Additional Information

**How to cite this article**: Zhen, S. *et al*. First Comprehensive Proteome Analyses of Lysine Acetylation and Succinylation in Seedling Leaves of *Brachypodium distachyon* L. *Sci. Rep.* **6**, 31576; doi: 10.1038/srep31576 (2016).

## Supplementary Material

Supplementary tables

Supplementary figures

## Figures and Tables

**Table 1:** The overlap proteins that found both with acetylation and succinylation in seedling leaves of Bd21.

Protein	Protein name	Protein	Protein name
721606963	14-3-3-like protein A	357136151	glycine dehydrogenase (decarboxylating) 2
357163385	2-Cys peroxiredoxin BAS1	721663099	glycine--tRNA ligase 1
357147655	2-methylene-furan-3-one reductase	721668124	heat shock 70 kDa protein
357111449	30S ribosomal protein S14	721691498	histone H3.2
721675727	40S ribosomal protein S28	721663671	isocitrate dehydrogenase [NAD] catalytic subunit 5
357123316	50S ribosomal protein L18	721692015	isocitrate dehydrogenase [NAD] regulatory subunit 1
357112209	50S ribosomal protein L6	357144699	lactoylglutathione lyase
721672372	5-methyltetrahydropteroyltriglutamate--homocysteine methyltransferase 1-like	357132456	malate dehydrogenase 1
357166491	60S ribosomal protein L7a	357111487	oxygen-evolving enhancer protein 1
357156810	adenosylhomocysteinase-like	357111658	oxygen-evolving enhancer protein 2
721666975	ADP,ATP carrier protein	721652166	peptidyl-prolyl cis-trans isomerase
357124561	aldehyde dehydrogenase family 2 member B7	357131671	peptidyl-prolyl cis-trans isomerase
357166054	aminomethyltransferase	357135091	peptidyl-prolyl cis-trans isomerase CYP20-2
194033146	ATP synthase CF1 alpha subunit	721627179	peroxisomal (S)-2-hydroxy-acid oxidase GLO1
194033156	ATP synthase CF1 beta subunit	357133147	phosphoglycerate kinase
194033155	ATP synthase CF1 epsilon subunit	357137038	phosphoglycerate kinase
226741312	ATP synthase subunit b	357147757	phospholipid hydroperoxide glutathione peroxidase 1
357122954	ATP synthase subunit d	357137138	phosphoribulokinase
721662162	ATP synthase subunit d	194033149	photosystem I P700 chlorophyll a apoprotein A1
357146106	ATP synthase subunit gamma	357124847	photosystem I reaction center subunit II
721666815	ATP synthase subunit O	357113396	photosystem I reaction center subunit III
357123105	ATP-dependent zinc metalloprotease FTSH 1	357126063	photosystem II 22 kDa protein
721628014	beta-amylase	221272414	photosystem II CP43 reaction center protein
721642858	carbonic anhydrase, chloroplastic isoform X1	221272363	photosystem II CP47 reaction center protein
357114208	catalase-1	357112354	photosystem II repair protein PSB27-H1
721663986	chaperone protein ClpC1	721656187	polyphenol oxidase
357114085	chaperonin CPN60-1	357138529	probable histone H2A.2
357124424	chlorophyll a-b binding protein 1B-21	721621103	probable pyridoxal biosynthesis protein PDX1.1
721653899	chlorophyll a-b binding protein 8	357124584	protein CURVATURE THYLAKOID 1A
357157014	chlorophyll a-b binding protein CP26	357122407	protein THYLAKOID FORMATION1
357122377	chlorophyll a-b binding protein CP29.2	357137675	protein THYLAKOID FORMATION1
357121229	chlorophyll a-b binding protein	357134108	protein TIC 62
721674279	chloroplast stem-loop binding protein of 41 kDa b	721659745	protein TIC110
357122458	cytochrome b6-f complex iron-sulfur subunit	721659748	protein TIC110
194033161	cytochrome f	194033148	PSI p700 apoprotein A2
357132047	dihydrolipoyl dehydrogenase 1	357114147	putative aconitate hydratase
357134512	dihydrolipoyl dehydrogenase	357135418	pyruvate dehydrogenase E1 component subunit beta-4
721605698	elongation factor 1-alpha	194033207	ribosomal protein L2
721663915	elongation factor 2	194033185	ribosomal protein S3
357149925	elongation factor Tu	721611027	ribosome-recycling factor
357145900	enolase	218525917	Ribulose bisphosphate carboxylase large chain
357123995	enoyl-[acyl-carrier-protein] reductase [NADH] 1	721670938	ribulose bisphosphate carboxylase small chain PW9
357116174	ferredoxin-dependent glutamate synthase	357155405	ribulose bisphosphate carboxylase small chain PW9
357149112	ferredoxin--NADP reductase	357155664	ribulose bisphosphate carboxylase/oxygenase activase A, chloroplastic isoform X2
357110920	ferredoxin--NADP reductase	357167236	ruBisCO large subunit-binding protein subunit alpha
357126021	fructose-1,6-bisphosphatase	357110700	ruBisCO large subunit-binding protein subunit beta
357126392	fructose-bisphosphate aldolase cytoplasmic isozyme	357125896	sedoheptulose-1,7-bisphosphatase
357157399	fructose-bisphosphate aldolase	357116394	serine hydroxymethyltransferase 1
357123886	fructose-bisphosphate aldolase	721662584	serine--glyoxylate aminotransferase
721675948	GDP-mannose 3,5-epimerase 2	357134135	stromal 70 kDa heat shock-related protein
357148020	germin-like protein 8-14	357113565	succinate dehydrogenase [ubiquinone] flavoprotein subunit
357148595	glutamate-1-semialdehyde 2,1-aminomutase	357150216	succinyl-CoA ligase [ADP-forming] subunit beta
357111762	glutamate--glyoxylate aminotransferase 2	721639170	superoxide dismutase [Mn] 3.1
357166465	glutamine synthetase	357160604	thioredoxin M-type
357113924	glutathione reductase	357110873	transketolase
357144527	glyceraldehyde-3-phosphate dehydrogenase 1	357155874	translationally-controlled tumor protein homolog
357163943	glyceraldehyde-3-phosphate dehydrogenase A	357159648	triosephosphate isomerase
357114230	glyceraldehyde-3-phosphate dehydrogenase B	357134997	uncharacterized protein At5g02240-like
357146969	glycine cleavage system H protein	357159925	UTP--glucose-1-phosphate uridylyltransferase
721613385	V-type proton ATPase catalytic subunit A		
