# Supplementary material for: First Comprehensive Proteome Analyses of Lysine Acetylation and Succinylation in Seedling Leaves of Brachypodium distachyon L
Source: Sci Rep. 2016 Aug 12;6:31576. doi: 10.1038/srep31576 (PMC4981852; doi:10.1038/srep31576)
Supplement: Supplementary figures [file srep31576-s2.doc]

**First Comprehensive Proteome Analyses of Lysine Acetylation and Succinylation in** **the Seedling Leaves of *Brachypodium distachyon* L.**

Shoumin Zhen, Xiong Deng, Jian Wang, Gengrui Zhu, Hui Cao,

Linlin Yuan, Yueming Yan^*^

**Supplementary Data**

**Supplementary Table Legends**

**Table S1.** **The detail information of acetylated (Table S1-1) and succinylated (Table S1-2) sites, acetylated and succinylated proteins identified in the seedling leaves of Bd21 from nano-LC-MS/MS.**

**Table S2. The statistic analysis of the acetylated and succinylated sites in one protein.** The detailed information of one site, two sites, three sites, four sites, five sites, six sites, seven sites, eight sites, nine sites, ten sites, twelve sites, sixteen sites in one protein, respectively.

**Table S3. The detail information of the overlap peptides found to be both acetylated and succinylated in seedling leaves of Bd21**

**Table S4. The detail informations of GO annotation of the acetylated (Table S4-1) and succinylated (Table S4-2) proteins.**

**Table S5. The information of acetylated and succinylated proteins of GO, KEGG and domain enrichment.** Tables S5-1, 3, 5, 7, and 9 are the detail information of GO enrichment of biology process, cellular component, molecular function, KEGG pathway enrichment and domain enrichment of acetylated proteins. Tables S5-2, 4, 6, 8, and 10 are the detail information of GO enrichment of biology process, cellular component, molecular function, KEGG pathway enrichment and domain enrichment of succinylated proteins.

**Table S6. The number of motif enrichment analysis of the acetylated (Table S6-1) and succinylated (Table S6-2) proteins with Motif-X.**

**Table S7. The possibilities of the secondary structures of the acetylated (Table S7-1) and succinylated (Table S7-2) peptides.**

**Table S8. Conservation analysis of acetylated proteins compared with *Synechocystis*, *Arabidopsis thaliana*, Soybean and Rice (Table S8-1) and succinylated proteins in seedling leaves of Bd21 compared with rice, *H. sapiens*-Hela Cell, *E.coli* and Yeast (Table S8-2).**

**Table S9. New identified proteins in seedling leaves of Bd21 compared with previous studies.**

**Table S10. The identified acetylated proteins in *Arabidopsis thaliana* and rice could also be succinylated compared with Bd21 in seedling leaves.**

**Table S11. PPI networks of some important acetylated (Table S11-1) and succinylated (Table S11-2) proteins by STRING.**

**Table S12. The detail information of the identified protein bands by western blotting identified by LC-MS/MS.** Table S12-1-5 are the detail information of the identified protein bands 1, 2, 3, 4 and 5 by western blotting identified by LC-MS/MS.

**Supplementary Figure Legends:**

**Supplemental Fig. S1**. **Strategy for a large-scale acetylated and succinylated proteomics study on seedling leaves of *B. distachyon.*L.**

**Supplemental. Fig. S2**. **Sequence alignment, three-dimensional structure, and tandem mass spectrometry (MS/MS) spectra map for DLD in the seedling leaves of Bd21.** **(a):** DLD sequence alignment with Bd21, *E.coli*, *S. cerevisiae* and *H. sapiens*. The red box showed the succinylated and acetylated sites of DLD. **(b):** Three-dimensional structure of DLD (confidence level >90%). **(c):** Succinylated peptide MS/MS spectra map shows the succinylated sites at Lys-405.

**Supplemental. Fig. S3. Sequence alignment, MS/MS spectra map and three-dimensional structure for three GAPDHs in the seedling leaves of Bd21.** GAPDH sequence alignment with soybean, *Arabidopsis*, rice and *Synechocystis* sp. PCC 6803 of protein **(a)** gi357114230, **(b)** gi357144527 and **(c)** gi354163943, respectively. The red box showed the sites with both two PTMs in GAPDH. Three-dimensional structure of GAPDH (confidence level >90%).

**Supplemental. Fig. S4**. **Protein−protein interaction (PPI) network of acetylated (a) and succinylated (b) proteins in different functions.** Different colours mean different function groups in acetylated and succinylated proteins. Ten groups were indentified in acetylated proteins and eight groups were showed in succinylated groups. In the network of succinylated proteins, ubiquitin-like and 14-3-3-A proteins connect with many other proteins.

**Supplemental. Fig. S5. The physiological changes of four periods of seedling leaves in Bd21.** (**a)** is the development picture of different periods. **(b):** The plant height of different periods of seedling leaves in Bd21. **(c):** The leaf length of four periods in seedling leaves in Bd21. **(d):** The root length of four periods in seedling leaves in Bd21. **(e):** The water content of the several periods in seedling leaves in Bd21. The plant height, leaf length and root length were measured with five replicates and the water content also tested with five replicates to insure the accuracy.


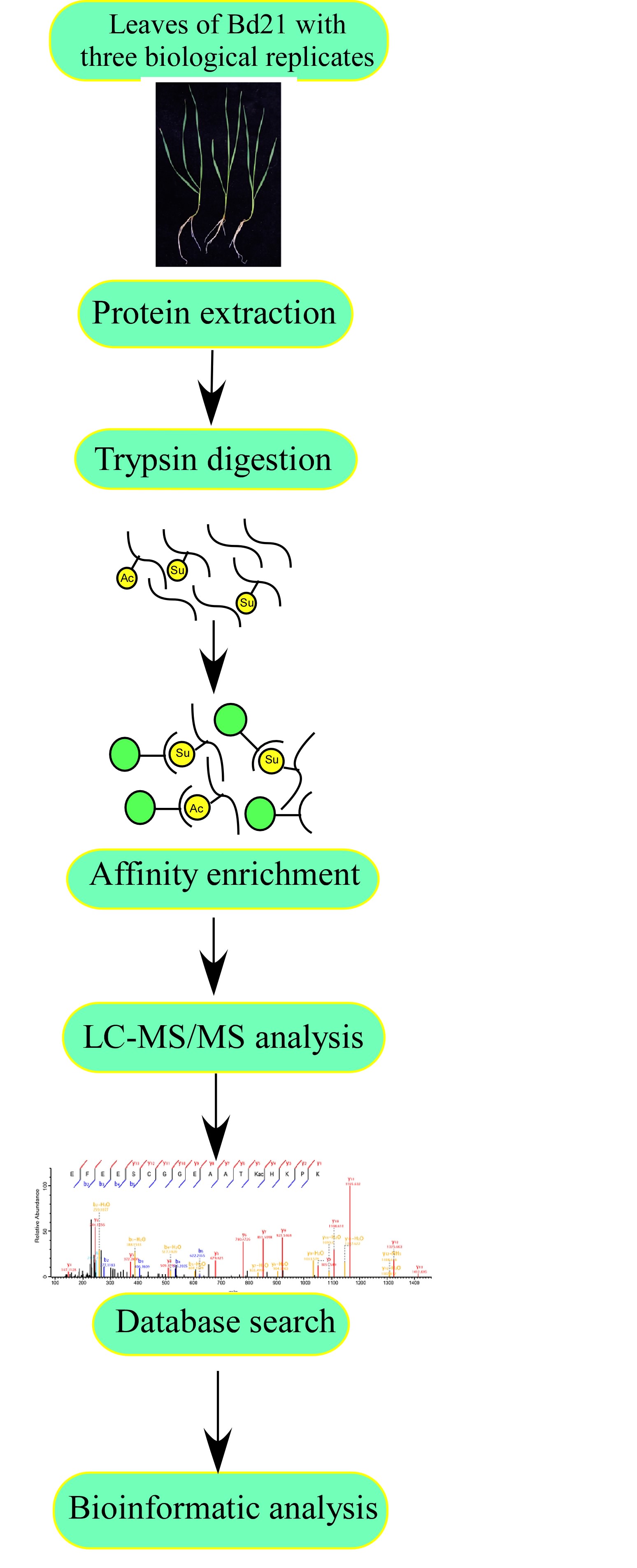


**Fig. S1**

**
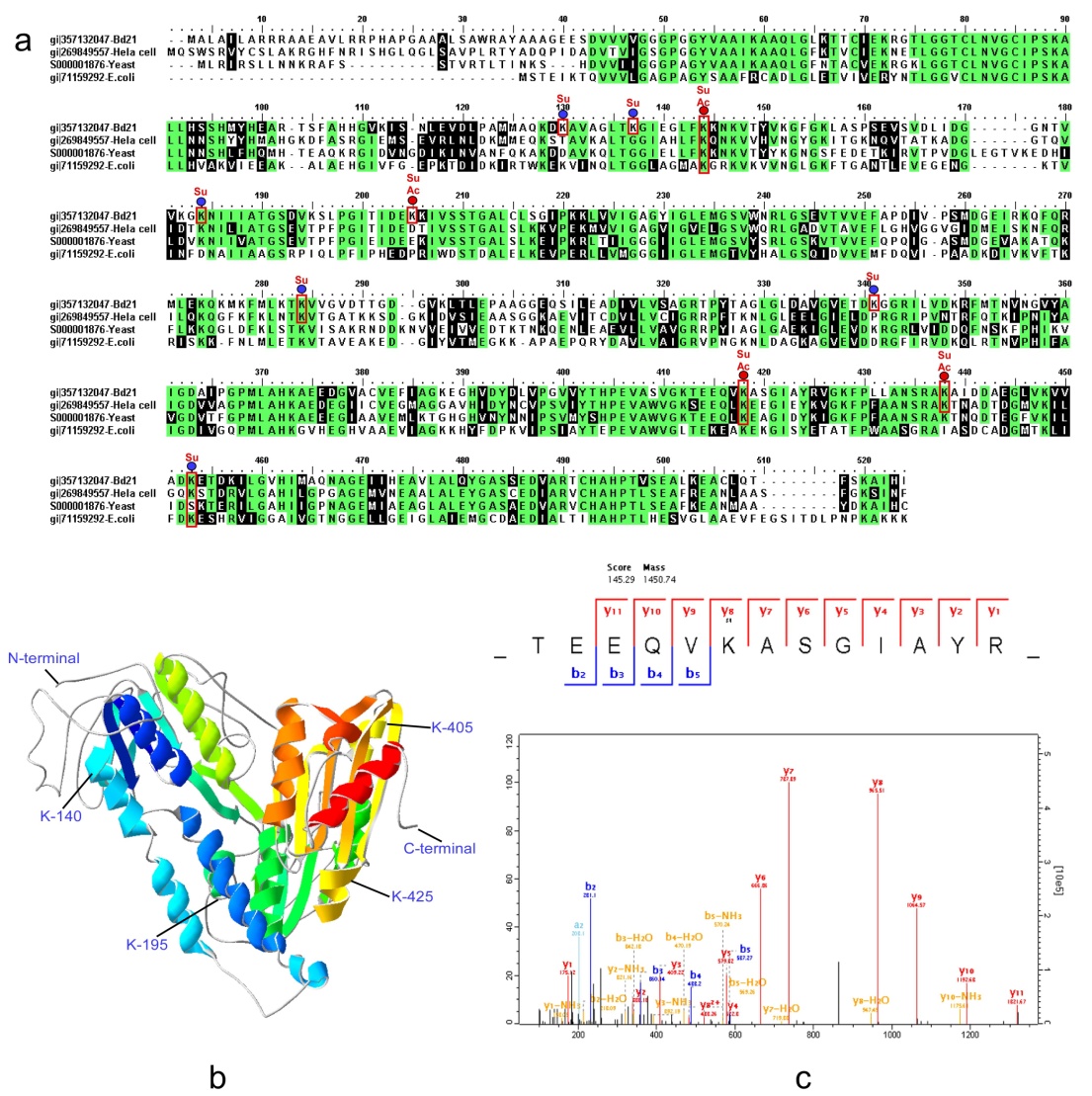
**

**Fig. S2**

**
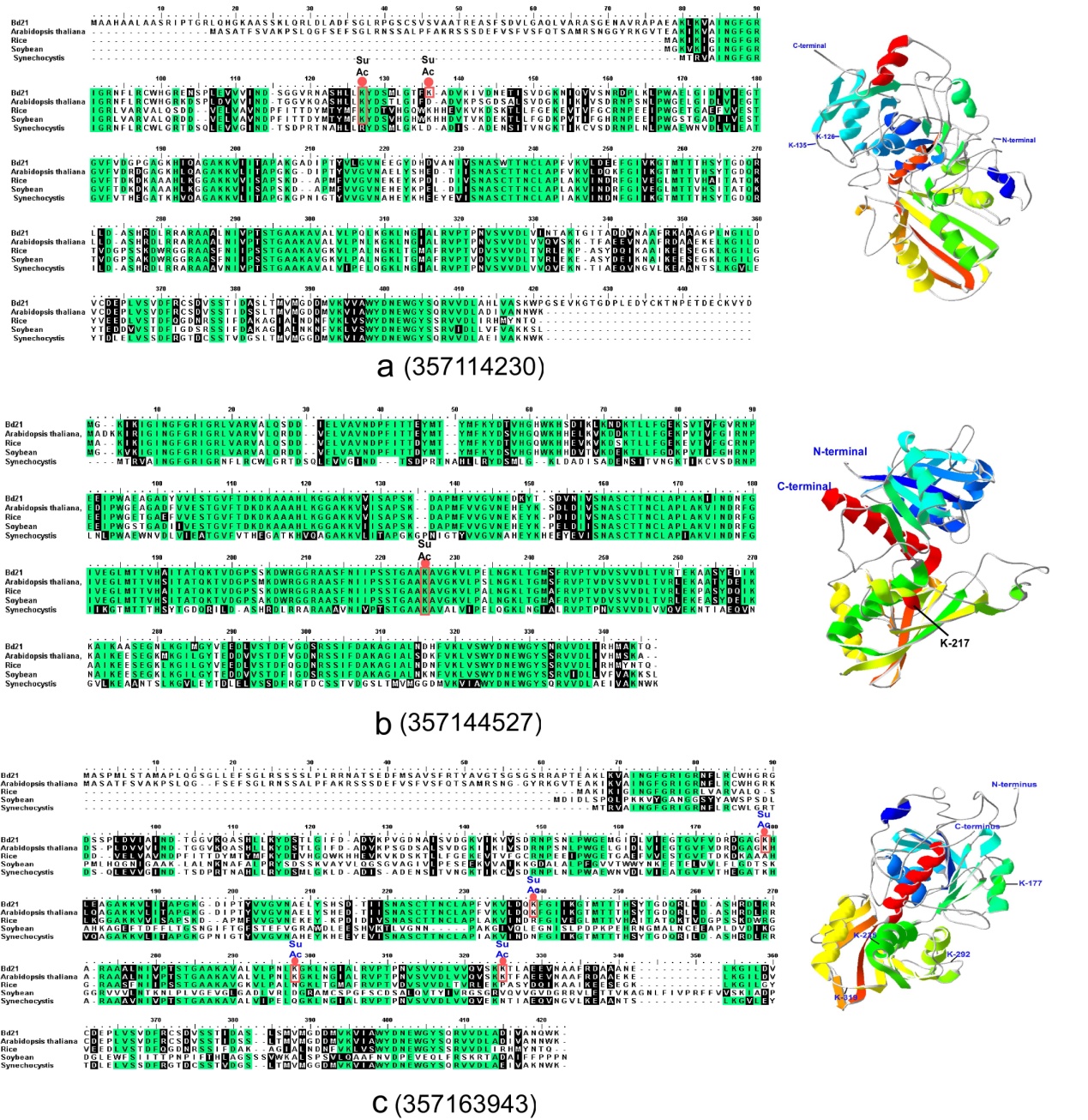
**

**Fig. S3**

**
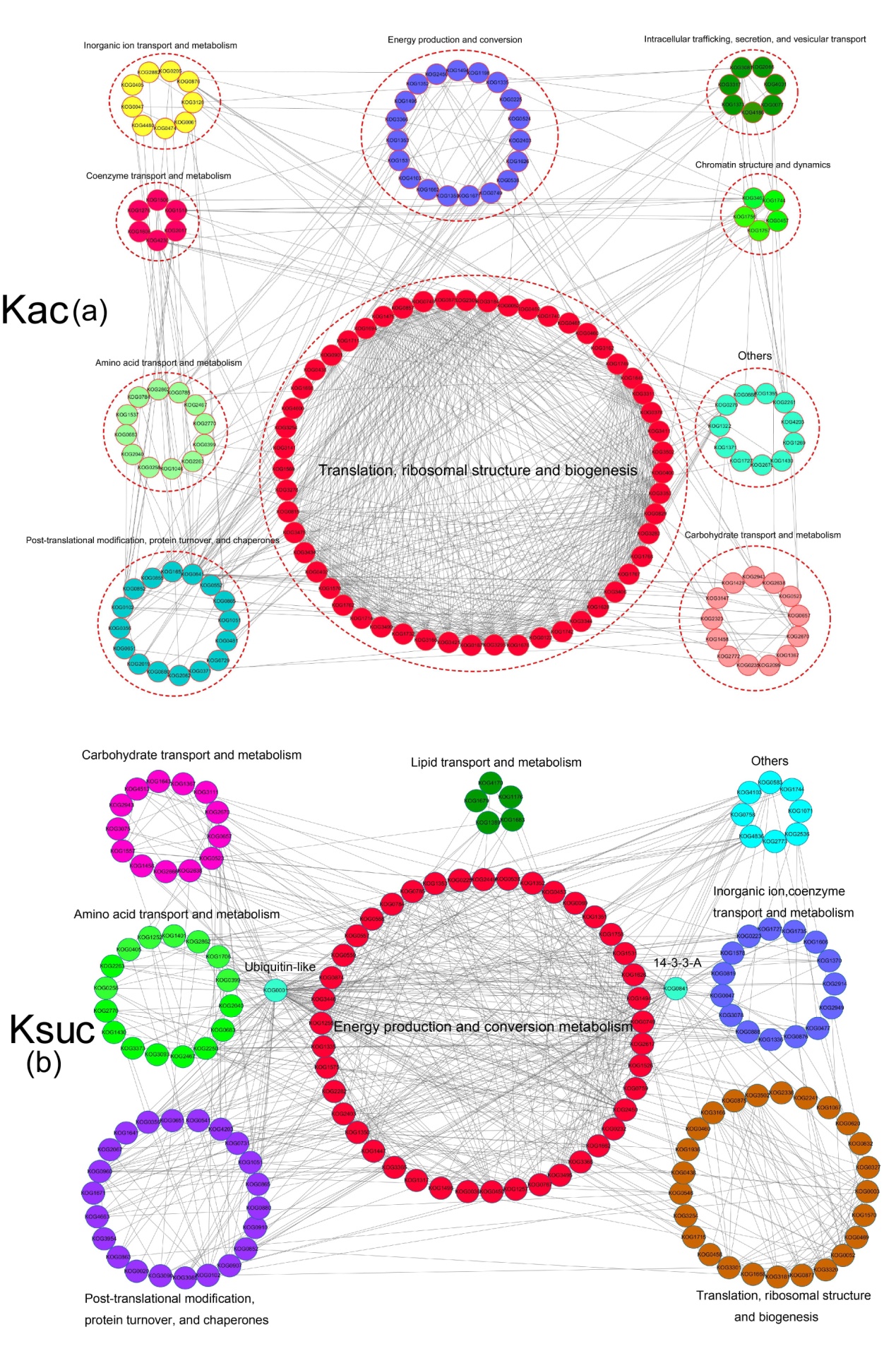
**

**Fig. S4**

**
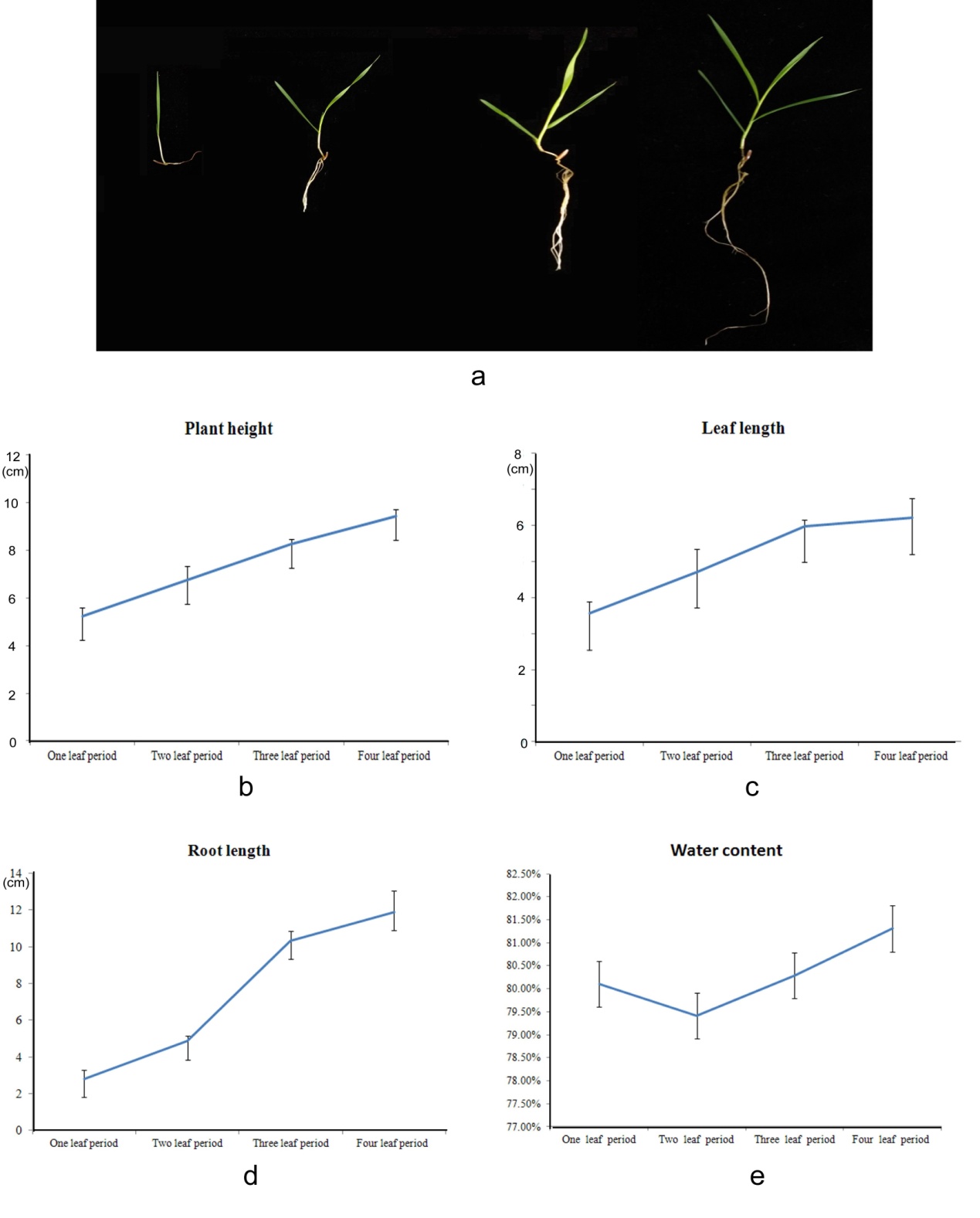
**

**Fig. S5**
